# Ecologic Immunology of Avian Influenza (H5N1) in Migratory Birds

**DOI:** 10.3201/eid1308.070319

**Published:** 2007-08

**Authors:** Thomas P. Weber, Nikolaos I. Stilianakis

**Affiliations:** * Joint Research Centre, Ispra, Italy; † University of Erlangen-Nürnberg, Erlangen, Germany

**Keywords:** Avian influenza, H5N1, avian migration, ecologic immunology, migratory birds, immunocompetence, immunosuppression, exercise, stress, perspective

## Abstract

The claim that migratory birds are responsible for the long-distance spread of highly pathogenic avian influenza viruses of subtype H5N1 rests on the assumption that infected wild birds can remain asymptomatic and migrate long distances unhampered. We critically assess this claim from the perspective of ecologic immunology, a research field that analyzes immune function in an ecologic, physiologic, and evolutionary context. Long-distance migration is one of the most demanding activities in the animal world. We show that several studies demonstrate that such prolonged, intense exercise leads to immunosuppression and that migratory performance is negatively affected by infections. These findings make it unlikely that wild birds can spread the virus along established long-distance migration pathways. However, infected, symptomatic wild birds may act as vectors over shorter distances, as appears to have occurred in Europe in early 2006.

## Abstract 2

Studies do not support the claim that migratory birds can spread highly pathogenic avian influenza (H5N1) over long distances.

Since its appearance in 1996 in a domestic goose in Guangdong Province, People’s Republic of China, highly pathogenic avian influenza (HPAI) caused by a virus of subtype H5N1 has repeatedly been portrayed as the most prominent emerging disease threat faced by humanity. In addition to its high mortality rate for infected humans (currently 60%), a worrisome aspect of Asian lineage HPAI (H5N1) is its rapid spread from East Asia to Central Asia, Europe, and Africa in 2005–2006. In 2006–2007, Southeast Asia remained the geographic center of outbreaks in animals and humans. Migratory birds as well as trade involving live poultry and poultry products have been suggested as the most likely causes of dispersal of the virus (*1*–*3*). Several outbreaks in Central Asia and Europe of HPAI (H5N1) among wild bird populations that were apparently not in contact with domestic birds led to an increased interest in the potential role of wild migratory birds in the long-distance dispersal of the virus.

Despite intensive research, the means by which this spread was accomplished have remained extraordinarily controversial. The divisiveness of this issue illustrates the point that an evaluation of emerging disease threats requires a broad interdisciplinary approach (*4*). It is thus disappointing that ornithologic knowledge and methods have not figured prominently in many high-profile studies that have shaped scientific, public, and political perceptions of the threat posed by HPAI (H5N1). Premature verdicts can have serious consequences. The view that disease transmission between wild birds and domestic poultry and humans is likely can seriously undermine conservation efforts concerning threatened migratory birds by eroding tolerance of what the public is led to believe are potential disease reservoirs.

We agree with Yasué et al. (*5*), who considered data on which migratory birds are considered responsible for long-distance spread of HPAI (H5N1) to be incomplete, inadequate, and often incorrect. For example, in a large number of cases involving wild birds in 2005 and early 2006, the Organisation Mondiale de la Santé Animale (Paris, France) did not report the species concerned. Lack of knowledge of the species involved in outbreaks among wild birds is just the tip of the iceberg. Even if species, age, and sex of affected birds were recorded correctly, many other interpretative issues often emerge. The ecology of infectious diseases and the immune system is an innovative field that has stimulated the attention and interest of ecologists (*6*) but is still struggling to be appreciated by the biomedical community. The field relies on fundamental information on the natural history and evolutionary ecology of the pathogens and hosts involved. Work on the natural history of avian migrants is published mainly in journals that easily escape the attention of veterinarians, virologists, epidemiologists, and molecular biologists. Relevant findings published in ecologic or physiologic journals are also easily missed by the scientists who deal most closely with avian influenza. An additional problem is that many important phenomena in avian movements are not well researched, e.g., movements caused by cold weather and migratory connectivity.

Yasué et al. (*5*) and Feare and Yasué (*7*) have reported numerous problems with the soundness of many results concerning the involvement of wild birds in the spread of avian influenza. We complement these criticisms by concentrating on the neglected topic of seasonal (and shorter term) variation in the physiology of bird migration and consider how this variation might affect and be affected by immunocompetence. The immune function of migratory birds has so far received little attention in relation to avian influenza. We present pertinent and representative findings in this field. We argue that the considerable physiologic stresses of long-distance flights cast some doubts on the assumption that migratory birds are capable of spreading HPAI (H5N1) on a continental and transcontinental scale.

## Ecologic Immunology of Migratory Birds

The hypothesis that migratory birds can transport HPAI (H5N1) over long distances rests on the assumption that some infected, virus-shedding wild birds show no or only mild symptoms and migrate long distances unhampered. There has been no direct test of this assumption, but several findings from ecologic immunology and exercise physiology studies are not compatible with this conjecture.

The immune system operates in a complex physiologic and ecologic context. The hormonal and nutritional states of an animal influence the functions of the immune system (*8*,*9*). These states are, in turn, affected by ecologic factors such as food supply, density of competitors and predators, energy expenditure, and injury. The fundamental idea of ecologic immunology is that maintaining a responsive immune system and mounting an immune response are energetically and nutritionally costly and that these costs have to be balanced against other expenses, such as reproduction, molting, growth, and development, that contribute to an animal’s fitness (*6*,*10*). Thus, it is not only the direct negative effects of parasites that determine the consequences of an infection, but also the costs of the immune response. These costs are likely to become visible in situations in which animals are resource-limited. Animals might, for example, allocate more resources to immune function if challenged by an infection and expend less energy in other activities. Caring for young is energy-demanding, and activation of the immune response during breeding results in lower reproductive success or parental effort (*11*). Birds give up some of their current reproductive success to safeguard their survival and expected future reproductive success. Activating the immune system without being challenged by parasites can be costly. In a laboratory experiment with bumblebees (*Bombus terrestris*), Moret and Schmid-Hempel (*12*) showed that activation of the immune system of starved bumblebees resulted in lower survival rates. Hanssen et al. (*13*) reported similar results with eiders (*Somateria mollissima*, a migratory sea duck).

Long-distance migration is one of the most demanding physiologic activities in the animal world, and an adaptive resource allocation between concurrent physiologic processes likely occurs. Birds migrate for hours or even days at extremely high metabolic rates. During long flights, they can sustain up to 10× the basal metabolic rate. The bar-tailed godwit (*Limosa lapponica baueri*) may fly 6,000–8,600 km nonstop from New Zealand to stopover locations in Southeast Asia (*14*). Ducks generally travel shorter distances between stopover sites. However, because of their heavier bodies and shorter wings, ducks are less dynamically efficient and probably experience physiologic stress during their shorter migratory flights. The periods between flights are sometimes called resting phases, but this is clearly a misnomer. These are periods of frantic energy acquisition and physical recovery. During these stopovers, birds increase their body weight by 30%–50% of their lean mass in a few days with mainly fat to fuel the next step in their journey. Birds have evolved physiologic and behavioral adaptations to deal with these extreme demands of both energy expenditure and acquisition. Birds, especially those that migrate between widely separated stopover sites, adjust to these demands by regularly and repeatedly rebuilding their bodies. They increase the size of the digestive system and decrease flight muscle mass in refueling periods, and they go through the opposite adjustments before departure (*15*).

Migratory birds are well-adapted feeding and flying machines, but the exertion involved still takes its physiologic toll. Guglielmo et al. (*16*) reported that migratory flights result in muscle damage. Macrophages and other phagocytic cells invade the injured muscle cells and remove them. Migration and channeling of resources from the immune system can release latent infections in songbirds (*17*). Figuerola and Green (*18*) showed that the number of parasite species or genera reported per migratory waterfowl host species is positively related to migration distance. However, to infer that birds that migrate long distances are affected disproportionately by parasites, it would be necessary to show that they host more parasite species from each geographic region they pass through than resident waterfowl from the respective region.

Migratory birds have also evolved mechanisms to cope with a greater diversity of parasites than resident species. Møller and Erritzøe (*19*) found that migratory birds have larger immune defense organs than closely related nonmigratory birds. Owen and Moore (*20*) showed that 3 species of thrushes migrating through mainland America (only flying at night and resting and feeding during the day) are immunocompromised during spring and autumn migration. In humans, postexercise immune function depression is most pronounced when exercise is continuous, prolonged, of moderate-to-high intensity, and performed without food intake (*21*). However, whether similar mechanisms linking exercise and immune function also apply to birds is not known.

These representative studies demonstrate that physiologic demands of long-distance migration can suppress the immune system. Far less information is available, however, on 1 important aspect: how do infected birds perform during long-distance migration? Møller et al. (*22*) showed that barn swallows (*Hirundo rustica*) with large energy reserves maintain better immune function during migration, clear ectoparasites and blood parasites more effectively, and arrive earlier at breeding grounds (which is an important determinant of reproductive success) than birds with poor energy reserves. Some indirect evidence shows how exercise during migration, infection, and immune responses could interact. As mentioned, Hanssen et al. (*13*) demonstrated that in eiders, immune system activation can have severe negative consequences. These researchers injected females with 3 different nonpathogenic antigens (sheep erythrocytes, diphtheria toxoid, and tetanus toxoid) early in their incubation period. Mounting of a humoral immune response against these antigens decreased the return rate to the breeding grounds in northern Norway from 72% to 27%, which implied a high cost of the immune response. However, it is not clear from these results whether birds died during migration or during overwintering or whether the reduced return rate reflected only failure of birds to migrate back to their breeding grounds. Also, the demands of thermoregulation can be substantial. Liu et al. (*23*) reported correlations between sudden temperature decreases and activation of latent infection with influenza A virus.

The most direct evidence of interaction between demands of migratory flights and infections was reported by van Gils et al. (*24*). These authors found that Bewick’s swans (*Cygnus columbianus bewickii*) infected with low pathogenic avian influenza A viruses of the subtypes H6N2 and H6N8 performed more poorly in terms of foraging and migratory behavior than uninfected birds (including birds that had recovered from a previous infection). Infected birds had lower bite rates, took more time to deposit the energy reserves required for migration, departed later, and made shorter journeys. The researchers suspect that the swans might have traded off energy invested in immune defense against energy invested in rebuilding their bodies for efficient fuel deposition and flight. However, as van Gils et al. (*24*) also reported, only a controlled experimental study can establish whether this hypothesis is plausible. However, such a study will probably never be done because release of the H5N1 subtype of HPAI virus into the wild is banned. A large number of studies of domestic and laboratory mammals show that many bacterial, viral, and parasitic infections lead to anorexia in the host (*25*). The findings reported by van Gils et al. (*24*) are consistent with known patterns of infection-induced anorexia in mammals.

These findings do not offer a definite rebuttal, but they cast some serious doubts on the frequently repeated claim that wild birds can easily act as long-distance vectors for influenza A viruses. However, some caveats need to be addressed that make any quick judgment impossible. The study by van Gils et al. (*24*) had a low sample size of infected birds. Furthermore, it was conducted during spring migration. In many migratory species, spring and autumn migration are likely to occur under different conditions. The considerable stress of spring migration may be amplified by energetically costly flights undertaken when food resources are often still scarce along the migratory route, as well as at breeding grounds at the time of arrival (*26*,*27*). After arrival at breeding grounds, the birds’ energy must be invested in display and, in females, in egg production. In autumn, feeding conditions are generally better along migratory route. If autumn migration, when infections with influenza A viruses are more prevalent in waterfowl, proceeds under more benign feeding conditions, the immune system of birds might be able to clear infections more effectively. This may mean that the birds can clear infections quickly or that the infection is controlled by the immune system but not entirely cleared, and virus-shedding still occurs. Hasselquist et al. (*28*) showed in a wind-tunnel experiment with the red knot (*Calidris canutus*), a long-distance migratory bird, that long flights did not influence immune responses. However, they also found that some birds with low antibody responses against tetanus refused to fly. This suggests that there is a trade-off between the demands of different physiologic systems and that only birds in good condition with energy to spare may be willing to expend this energy.

Sparse findings on immunocompetence and exercise in migratory birds do not decisively rule out the possibility that HPAI (H5N1) may be transported relatively short distances by wild birds. That birds are leaving areas with cold weather does not necessarily imply stressful long flights and the physiologic adjustments that accompany long-distance migration. Even birds incapacitated by an infection may therefore manage to escape harsh weather. However, causes and consequences of cold weather movements have not been investigated in sufficient detail (*29*). An analysis by Feare (*30*) supported the view that long-distance spread of virus by migratory birds is unlikely but short-distance spread is possible. Feare (*30*) examined all known major outbreaks in wild birds and concluded that most occurrences reflect local acquisition from a contaminated source, followed by rapid death nearby. Outbreaks in Europe in 2006 indicate that infected wild birds can travel a limited distance before dying of influenza and can pass the virus to other wild or domestic birds.

## Conclusion

No convincing evidence has yet shown that infected, asymptomatic wild birds can or do carry influenza virus along established, seasonal long-distance migration routes. Even infected dying swans do not shed HPAI (H5N1) in large quantities; swans may thus constitute an end host and not be carriers or efficient transmitters (*31*,*32*). The controversies surrounding HPAI (H5N1) and its likely mode of spread show how little is known about some important topics in the field of emerging infectious diseases. These topics include epidemiology of parasites with highly mobile host species and function of the immune system of these highly mobile host species who experience diverse climatic and ecologic conditions and variable parasite faunas during their annual cycle.

Recent work on the role of migratory Saiga antelopes in livestock disease epidemiology has shown how host movement, multiple host species, and temporal and climatic variation must be included in population dynamics models of parasites (*33*). However, studies must go beyond such necessary and welcome modeling efforts. Research in ecologic immunology has shown that the functionality of the immune system has to be considered in an ecologic and evolutionary life-history context. The immune system shows complex and, from an evolutionary point of view, often adaptive dynamics with multifaceted interactions with nutritional, hormonal, and energetic states and other physiologic processes. However, ecologic immunology is a discipline in its infancy and still often works with rather simplistic ideas. For example, the immune system is often implicitly assumed to be a unified system that competes with other physiologic processes for energy and nutrients. Long and Nanthakumar (*34*) showed this to be an unrealistic and naive assumption; they emphasize the necessity of considering the differential effects of energy or nutrient stress on specific subcomponents of the immune system.

It therefore remains a critical task to research the capacities and limitations of the immune system in wild birds under natural conditions. Only then will it be possible to judge how results from laboratory experiments can be transferred to natural situations. For example, Hulse-Post et al. (*35*) have shown that HPAI (H5N1) evolves to lowered pathogenicity in captive laboratory-maintained mallards (*Anas platyrhynchos*) but remains highly lethal for chickens. This finding suggests that ducks may act as asymptomatic carriers. However, it remains unclear whether free-living, migratory wild ducks facing stressors such as food shortages or long flights are as immunocompetent as their laboratory counterparts or whether virus evolution takes the same course under such conditions. The commercial movement of asymptomatically infected domestic ducks, often for pest control reasons and over longs distances, could be a mechanism of spread.

Two of the major challenges in the 21st century are emerging diseases and the protection of biodiversity. Sustainable solutions for these challenges can be fostered only in a respectful interdisciplinary atmosphere. Migratory birds are already affected by habitat destruction and climate change; alarmist statements blaming migrants for the spread of an emerging disease with pandemic potential and ignoring or underplaying the role of the poultry industry do not do justice to the complexity of the issues involved (*36*,*37*).
